# Purification of polyclonal anti-conformational antibodies for use in affinity selection from random peptide phage display libraries: A study using the hydatid vaccine EG95

**DOI:** 10.1016/j.jchromb.2009.03.036

**Published:** 2009-05-15

**Authors:** A.J. Read, C.G. Gauci, M.W. Lightowlers

**Affiliations:** The University of Melbourne, Veterinary Clinical Centre, 250 Princes Highway, Werribee, Victoria 3030, Australia

**Keywords:** GST, glutathione S-transferase, HRP, horseradish peroxidise, IPTG, isopropyl-1-thio-β-galactoside, MBP, maltose binding protein, PBST, phosphate buffered saline-Tween®, PBS, phosphate buffered saline, RPPD, random peptide phage display, SMPBS, skim milk phosphate buffered saline, TMB, tetra-methylbenzidine, Phage display, Monospecific antibodies, *Echinococcus granulosus*

## Abstract

The use of polyclonal antibodies to screen random peptide phage display libraries often results in the recognition of a large number of peptides that mimic linear epitopes on various proteins. There appears to be a bias in the use of this technology toward the selection of peptides that mimic linear epitopes. In many circumstances the correct folding of a protein immunogen is required for conferring protection. The use of random peptide phage display libraries to identify peptide mimics of conformational epitopes in these cases requires a strategy for overcoming this bias. Conformational epitopes on the hydatid vaccine EG95 have been shown to result in protective immunity in sheep, whereas linear epitopes are not protective. In this paper we describe a strategy that results in the purification of polyclonal antibodies directed against conformational epitopes while eliminating antibodies directed against linear epitopes. These affinity purified antibodies were then used to select a peptide from a random peptide phage display library that has the capacity to mimic conformational epitopes on EG95. This peptide was subsequently used to affinity purify monospecific antibodies against EG95.

## 1 Introduction

**Fig. 1:**
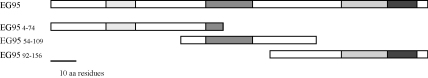
Schematic representation of full length recombinant EG95 and three overlapping truncated versions of EG95. Linear epitope regions identified by Woollard et al., [3] are shaded.

Hydatid disease is a zoonotic parasitic disease with a pan-global distribution. It results in considerable human morbidity and mortality, particularly in developing countries. The disease also occurs in economically important mammals such as sheep and cattle. Hydatid disease is caused by the metacestode stage of the canine tapeworm *Echinococcus granulosus*. A recombinant vaccine known as EG95 has been developed that can be used to prevent hydatid infection in animal intermediate hosts [1,2]. The EG95 vaccine comprises a single recombinant antigen and much progress has been made with its characterisation. It induces complement-fixing antibodies that kill the invading oncosphere early in an infection. Woollard et al. [3] discovered four linear epitopes on EG95 using overlapping synthetic peptides. However, these epitopes were found to be of a minor specificity in the total immune response and when they were used as an immunogen, they were not able to elicit protective antibodies [4]. Woollard et al. [5] also produced three overlapping polypeptides that span the length of EG95. These polypeptides contained all the previously discovered linear epitopes, but each lacked the tertiary structure of EG95. These polypeptides failed to protect sheep from hydatid disease when used as an immunogen. A schematic representation of these polypeptides, and their relationship to the identified linear epitopes and full length EG95 is shown in Fig. 1. Since linear epitopes do not protect against infection, it remains that the protective epitopes of EG95 are conformational in nature and the entire length of the EG95 molecule is required to produce immunity [5]. The current study is aimed towards identification of peptides that have the potential to immunologically mimic one or more of these conformation epitopes.

Random peptide phage display (RPPD) libraries contain large numbers of phages that display different peptides of random amino acid sequence. These random peptides are capable of binding to a wide variety of ligands in a process known as affinity selection. When these libraries are used to identify peptides that mimic antigen epitopes, the most common ligand used is antibody. The majority of published papers describing the use of antibody as the basis for affinity selection have used monoclonal antibodies. Monoclonal antibodies have certain desirable properties which make them the usual choice in epitope/mimotope selection experiments when compared to polyclonal antibodies. These include their homogeneity that results in the ability to treat the antibody as a defined chemical, and their precise specificity that identifies a single epitope. The homogeneity and precise specificity of monoclonal antibodies mean that peptides selected using phage display can be assessed for affinity to the antibodies and the results are not confounded by the inclusion of peptides that mimic more than one epitope. Monoclonal antibodies have been used to clearly identify peptides that mimic both linear and conformational epitopes [6,7].

However, there are also limitations in using monoclonal antibodies. First, monoclonal antibodies are more labour intensive and expensive to produce when compared to polyclonal antibodies. Furthermore, a monoclonal antibody can only be a useful target if there is evidence that it recognises an epitope that has a protective function *in vivo*. Epitopes recognised by monoclonal antibodies may not be protective because some may be of minor specificities, while others may not be accessible to the immune system because of glycosylation or other conformational factors [8]. Polyclonal antibodies, on the other hand, target multiple epitopes and so contain specificities that cover the full range of epitopes on an antigen that are presented to the immune system. Polyclonal antibodies typically have higher avidity for the antigen in question [9] and are more tolerant of minor changes in the antigen than monoclonal antibodies, for example differences in glycosylation, or mild denaturation [10].

Preliminary experiments using affinity selection of random peptides on polyclonal antibodies from sheep that have been previously vaccinated with EG95 were performed [11]. Interestingly, this affinity selection resulted in a large number of clones exhibiting a peptide insert sequence with a strong consensus motif that corresponded to one of the linear epitopes of EG95 discovered by Woollard et al. [3]. This linear epitope was shown to be of minor specificity and not to be protective.

This result highlights a bias that appears to be inherent in the use of polyclonal antibodies as the target for RPPD. Craig et al. [12] first alluded to this bias. They used 15 different RPPD libraries to pan polyclonal antibodies to either hen eggwhite lysozyme or worm myohemerythrin and found that all 102 peptides that they selected could be aligned to linear epitopes on either protein. This is despite the majority of antibodies present in a polyclonal sample being directed against conformational epitopes [13,14].

The number of studies that use polyclonal antibodies as the basis for affinity selection of random peptides is relatively few. Only two studies [15,16] found peptides that mimic only conformational epitopes. With the exception of Iniguez et al., [17], all other studies (where the amino acid sequence of the target was known) found that the majority of selected peptides could be aligned to linear epitopes [12,15,16,18–38]. Hence when RPPD technology is applied to polyclonal antibodies, it is likely that there is an inherent bias toward the selection of peptides that mimic linear epitopes. If RPPD libraries are to be used for the discovery of peptides that mimic conformational epitopes using polyclonal antibodies, then a method must be devised to eliminate this bias.

The intent of this study is to present a method for affinity purification of polyclonal antibodies against EG95 that are depleted of linear epitope specificities. These anti-conformational antibodies are then used to discover peptides that can mimic the conformational epitopes of EG95. Further details on the evaluation of these peptides can be found elsewhere [39].

## 2 Materials and methods

### 2.1 Production of GST and GST fusion proteins in *Escherichia coli*

Eight recombinant proteins were used for immunisation, affinity purification and ELISAs. The derivation of vectors and the expression of these proteins have been detailed elsewhere [1,3,40]. Briefly, glutathione S-transferase (GST) and fusion proteins containing three, non-protecting, truncated and overlapping derivatives of EG95 were expressed from the pGEX-3-EX expression plasmid (Amersham Pharmacia Biotech, Sweden) in *E. coli* JM109 strain (New England Biolabs, USA). The full length recombinant EG95 was also expressed from the pGEX-3-EX expression plasmid and from the pMAL-C2 (New England BioLabs, USA) plasmid as a maltose binding protein (MBP) fusion in *E. coli* BB4 cells (Stratagene, USA). GST and GST fusion proteins were purified from isopropyl-1-thio-β-galactoside (IPTG) induced bacterial cell cultures by glutathione-agarose affinity chromatography as described by Smith and Johnson [41]. MBP fusion proteins were affinity purified on amylose resin (New England BioLab, USA) according to the manufacturer's recommendations. The three truncated proteins were designated EG95_4–74_, EG95_54–109_, and EG95_92–156_, subscripts referring to the location of the amino acid residues in the translated native protein. A graphical representation of these proteins is shown in Fig. 1.

### 2.2 Immunisation of experimental animals

Two 3-year-old Merino wethers were used for the production of anti-EG95–GST antibodies. Immunisation occurred on three occasions with 100 μg of EG95–GST and 1 mg of Quil-A (adjuvant) in 2 ml of phosphate buffered saline (145 mM NaCl, 2.7 mM KCl, 12 mM Na_2_HPO_4_, 1.2 mM KH_2_PO_4_, pH 7.4) (PBS). The second immunisation took place 14 days after the initial immunisation, and the third immunisation 45 days after the initial immunisation. Serum was collected at day 59.

### 2.3 Preparation of affinity purification columns

A total of six protein affinity purification columns were produced. GST, EG95_4–74_–GST, EG95_54–109_–GST, EG95_92–156_–GST, *E. coli* lysate and EG95–GST were ligated to agarose beads via CNBr linkage (CNBr-Sepharose, Amersham, Sweden). The fusion proteins were prepared for the linking reaction by membrane filtration (0.2 μm pore size) (Minisart CE; Sartorius, Germany) and concentration using a 10 kDa concentrator (Vivaspin20, Sartorius, Germany). The buffer was exchanged to a coupling buffer (0.2 M NaHCO_3_, 0.5 M NaCl, 2 M urea, pH 8.3) using Vivaspin 20 Diafiltration Cups (Sartorius, Germany). Coupling of the proteins was performed according to manufacturer's guidelines. Briefly, 1 g of dried CNBr-Sepharose powder was allowed to swell in ice cold 1 mM HCl for 15 min. The beads were washed in 50 ml of ice cold 1 mM HCl. A final wash in coupling buffer was performed.

Concentrated protein and activated CNBr-Sepharose beads were mixed for 2 h at room temperature. The unbound protein was washed with two washes of coupling buffer. Remaining activated sites were blocked with three column volumes of glycine buffer (0.2 M glycine, pH 8.0) at 4 °C overnight. Alternating washes with coupling buffer followed by acetate buffered saline (0.1 M acetate, 0.5 M NaCl, pH 4.0) were performed four times in order to remove residual glycine and regenerate the column. Finally each column was equilibrated with three washes of PBS.

### 2.4 Affinity purification of anti-EG95 and anti-GST antibodies

#### 2.4.1 Depletion of unwanted antibody specificities

**Fig. 2:**
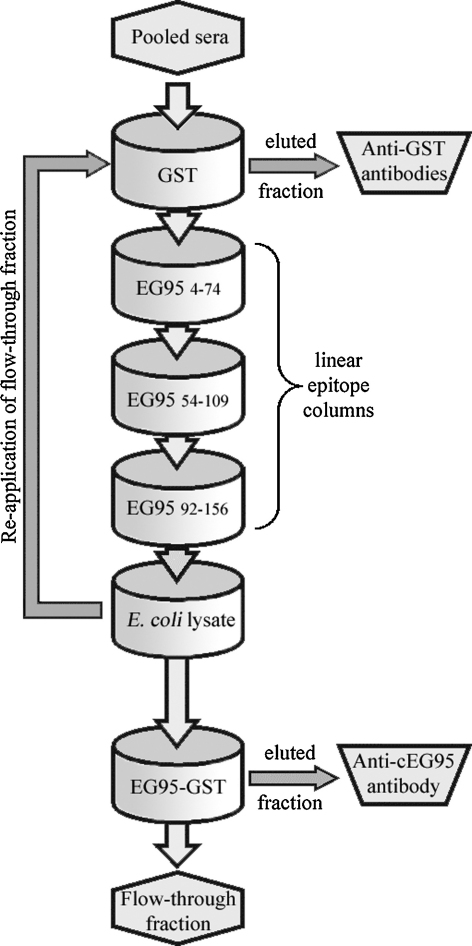
Schematic illustrating affinity purification technique. Clarified pooled antisera was diluted in binding buffer. The diluted serum was applied to the five depletion columns – GST, EG95_4–74_, EG95_54–109_, EG95_92–156_ and *E. coli* lysate. The flow through was reapplied to the depletion columns three times. Washed columns were eluted of antibodies. Anti-GST antibodies were collected. The flow through from the five depletion columns was applied to an EG95–GST column. The flow through from this column was reapplied three times. Washed EG95–GST column was eluted of antibodies and the antibodies against conformational epitopes on EG95 were collected (anti-cEG95).

Antisera from the immunised sheep were pooled, clarified and diluted with an equal volume of binding buffer (0.01 M sodium phosphate, 0.15 M NaCl, 0.01 M EDTA pH 7.0). Antisera were passed three times serially through the GST, EG95_4–74_, EG95_54–109_, EG95_92–156_ and *E. coli* lysate columns (Fig. 2). Columns were then washed with 10 column volumes of binding buffer. The wash solution and depleted antisera was concentrated using a 100 kDa concentrator (Vivaspin100, Sartorius, Germany) back to the original volume. The depleted serum was then passed three times over the EG95–GST column in order to capture anti-EG95 antibodies.

#### 2.4.2 Elution of affinity purified antibodies

The EG95–GST and GST columns were equilibrated with 10 column volumes of PBS. Antibodies were eluted in 1 ml fractions with a low pH buffer (0.1 M glycine/HCl, 0.15 M NaCl pH 2.6). Fractions were neutralised with 1 M Tris, pH 9.0. Individual fractions with optical density at 280 nm of greater than 0.2 were pooled and the total volume of this pool was concentrated using a 100 kDa concentrator (Vivaspin100, Sartorius, Germany) back to the original volume. The purified antibodies against conformational epitopes of EG95 are referred to as anti-cEG95, while the antibodies eluted from the GST column are referred to as anti-GST.

### 2.5 Enzyme linked immunosorbent assays (ELISA)

Determination of antibody titres was performed after Woollard et al. [3]. Microtitre plates (Greiner, USA) were coated with 50 μl of a 0.5 μg/ml solution of EG95–MBP in carbonate buffer (50 mM carbonate, pH 9.0) overnight at 4 °C. Wells were blocked for 1 h at room temperature with 100 μL of 5% skim milk in PBS (SMPBS). Test samples were incubated at room temperature for 1 h. Donkey anti-sheep horseradish peroxidise (Sigma, USA)) diluted 1:2000 in SMPBS was added for 1 h at room temperature. TMB substrate (0.09 M sodium acetate, 0.01 M citric acid, 0.417 μM 3,3′,5,5′-tetra-methylbenzidine, H_2_O_2_ (0.00036%, v/v)) was left to develop at room temperature for 30 min. The reaction was stopped by the addition of 0.5 M H_2_SO_4_. Absorbance values were read at 450 nm using an Opsys MR™ absorbance reader (Dynex Technologies, USA). The titre was defined as the reciprocal of the dilution that gave an absorbance reading of 1.0. Anti-GST antibody titres were determined in a similar manner except that a 1 μg/ml solution of GST was used to coat the microtitre plates. All titrations were performed in duplicate and results shown as mean.

### 2.6 Immunoblot assays

Nitrocellulose 0.2 μm (Hybond™ ECL™, GE Healthcare, USA) was pre-wetted in Towbin transfer buffer (25 mM Tris, 193 mM glycine, 20% methanol, pH 8.4). For Western assays, 1 μg of recombinant proteins was transferred from SDS-PAGE. For dot-blot assays, 1 μg of recombinant proteins EG95–GST, GST and EG95–MBP was spotted on to the nitrocellulose membrane. The membrane was subsequently dried and incubated for 1 h in SMPBST to block non-specific binding sites. Incubation with primary antibodies took place for 1 h at room temperature. Whole serum pools from sheep prior to, and after immunisation with EG95–GST, were diluted 1:2000 in SMPBST. Affinity purified anti-cEG95, anti-GST, anti-E100 and anti-G1 were diluted to 1 μg/ml. The primary antibodies were incubated for 1 h at room temperature with a 1:2000 dilution of polyclonal donkey anti-sheep IgG-HRP (Sigma, USA) conjugate in SMPBST. The antibodies on the paper were detected and visualised by Enhanced Chemiluminescence (ECL; Amersham Biosciences).

### 2.7 Affinity selection with random peptide phage display libraries

Two different phage display libraries were used, each expressing an unconstrained random peptide at the N-terminus of the pIII capsid protein. The PhD-12 phage Display Kit (New England Biolabs, USA) contains a library that expresses a 12 mer peptide with approximately 1 × 10^9^ unique random peptide inserts. The second library was a 20 mer library that expresses a 20 mer peptide with approximately 5 × 10^8^ inserts (supplied by AdAlta Pty Ltd., Australia).

Both the 12 mer and 20 mer libraries were subjected to four rounds of biopanning using anti-cEG95 antibodies as described by Read et al. [39]. The 20-mer library was biopanned in a similar manner using anti-GST antibodies in an attempt to discover a GST mimic that might be used as a control in further experiments.

### 2.8 Clone selection and DNA sequencing

Phages from the final round of panning were selected for sequencing. The DNA encoding the PIII random peptide insert was amplified by PCR using the following primers:

fd5’2 forward primer 5′-GTATTCTTTCGCCTCTTTC-3′
gIII 3’ reverse primer 5′-TGTAGGCATTCCACAGACAG-3′

Big Dye™ (Applied Biosystems, USA) termination sequencing was performed on the PCR products. Electrophoresis of the extension product was carried out at Micromon DNA Sequencing Facility (Monash University, Australia) using an Applied Biosystems model 3730 Sequencer.

### 2.9 Peptide synthesis

Peptides corresponding to the sequences of 13 of the phage clones selected by panning on anti-cEG95 antibodies, along with three of the phage clones selected by panning on anti-GST antibodies and one irrelevant peptide (R1) were synthesised using standard 9-fluorenylmethoxycarbonyl (FMOC) chemistry. Synthesis was performed manually and using an automated solid phase synthesiser (Symphony™ automated peptide synthesiszer, Rainin-PTI Woburn, USA) on a cleavable resin. Peptides were purified by reverse phase chromatography using Vydac C18 column (100 mm × 300 mm).

The ability of peptides bound to polyethylene glycol polyamide copolymer (PEGA) or mircotitre plates to react with antisera from sheep immunised with EG95–GST was assessed in a series of pilot studies (data not shown).

### 2.10 Affinity purification of monospecific antibodies

The result of the pilot studies indicated that peptide E100, HYKWLNDPLAAW, was potentially a mimotope for EG95 and suitable for use as an affinity ligand. Peptide G1, FSLYRVSGFDDPILFAMGPK, was chosen as an affinity ligand for antibodies directed against GST. NHS Sepharose 4 Fast Flow was obtained from Amersham Bioscience. E100 and G1 peptides were coupled to NHS-activated Sepharose 4B according to the manufacturer's instructions. Briefly, peptides were coupled at a concentration of 1.0 mg/ml for 3 h. Unbound sites were then blocked with Tris–HCl, pH 8.0 buffer. The peptide-affinity columns were washed successively with 0.1 M NaHCO_3_, 1 M Na_2_CO_3_, deionized water, 0.3 M glycine–HCl, pH 2.2, and PBS prior to use.

Pooled serum from sheep immunised with EG95–GST was diluted in an equal volume of binding buffer (0.01 M Na_2_HPO_4_, 0.15 M NaCl, 0.01 M EDTA, pH 7.0). The antisera solution was applied to the affinity columns four times. Unbound materials were removed by washing with six column volumes of binding buffer. The bound antibodies were eluted with low pH buffer (0.1 M glycine/HCl, 0.15 M NaCl pH 2.6). Fractions were neutralised with 1 M Tris, pH 9.0. The fractions with antibody ELISA absorbance values >0.75 were pooled. This pool was concentrated using a 100 kDa concentrator (Vivaspin100, Sartorius, Germany) back to the original volume. SDS-PAGE was used to confirm that only immunoglobulins had been purified and the antibody concentration was determined using the Biorad Protein Assay (Bio-Rad, USA). The antibodies eluted from the peptide E100 column are referred to as anti-E100, while the antibodies eluted from the peptide G1column are referred to as anti-G1.

### 2.11 Inhibition ELISA

The ability of peptides E100 and R1 to inhibit the binding of anti-E100 antibody to EG95–MBP was performed in a manner similar to the ELISA protocol described above. Peptide of varying concentrations was premixed with anti-E100 antibody for 1 h prior to addition of the antibody to the EG95–MBP coated wells. Results were expressed as absorbance at 450 nm.

## 3 Results

### 3.1 Antibody purification

Antisera from sheep immunised with EG95–GST were collected, clarified and pooled. The pooled antisera were shown to have a high titre against EG95–MBP. Affinity purification of the antisera involved the removal of antibody specificities to the fusion protein GST, as well as linear epitopes of GST and *E. coli* proteins. The removal of these specificities was attempted using protein specific columns. The antisera depleted of these specificities were then passed through an affinity column containing EG95–MBP. Purified antibodies were collected from this column. These antibodies directed against conformational epitopes on EG95 were designated the term anti-cEG95. A schematic representation of the affinity purification is shown in Fig. 2.

Coomassie stained SDS-PAGE showed that the affinity purified product was entirely made up of approximately 150 kDa proteins that, when reduced and alkylated, gave two products of approximately 25 and 50 kDa, respectively (data not shown).

**Fig. 3:**
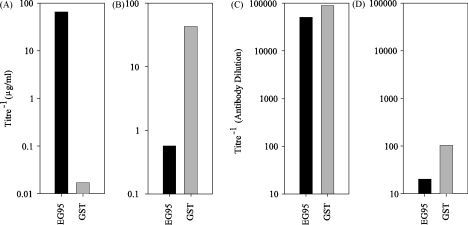
Comparison of the anti-EG95 and anti-GST titres of affinity purified antibodies. The EG95 (black bars) and GST (grey bars) reactivity of two affinity purified antibody solutions (anti-cEG95 and anti-GST). Panel A shows IgG affinity purified to conformational epitopes of EG95 (Anti-cEG95). Panel B shows IgG affinity purified to GST (Anti-GST). Panel C shows pooled whole antisera from sheep following third immunisation with EG95–GST (day 59). Panel D shows pooled whole antisera following removal of EG95 and GST determinants (flow-through fraction – see Fig. 2).

ELISA studies showed that the anti-cEG95 antibody solution was highly specific for EG95 (Fig. 3A). Likewise the anti-GST antibody solution was specific for GST (Fig. 3B). Specificity analysis revealed that the ratio of EG95–MBP titre to GST titre was over 2000 times greater in the anti-cEG95 antibody solution compared to the original pooled antisera. The ratio of GST titre to EG95–MBP titre in the anti-GST antibody solution was over 1000 times greater compared to the original pooled antisera. The titres of the pooled whole antisera and the pooled antisera following depletion of specific antibodies is shown in Fig. 3C and D, respectively.

### 3.2 Antibody specificity confirmation by dot-blot assay

**Fig. 4:**
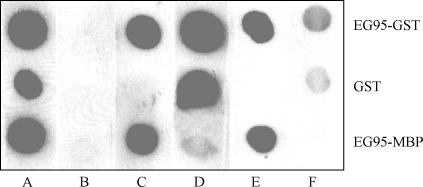
Dot-blot of recombinant protein antigens reacted with antibody pools from sheep immunised with EG95–GST. 1 μg of each protein was spotted onto the nitrocellulose membrane. Lane A is pooled serum from sheep immunised with EG95–GST. Lane B is the pre-immune pool. Lane C is reacted with IgG affinity-purified to conformational epitope/s of EG95 (anti-cEG95). Lane D is reacted with IgG affinity-purified to GST (anti-GST). Lane E is reacted with antibody affinity-purified to E100 peptide (anti-E100). Lane F is reacted with antibody affinity-purified to G1 peptide (anti-G1).

Dot-blot analysis (Fig. 4) confirmed the specific separation of the two antibody pools. Anti-cEG95 antibody recognised EG95–GST and EG95–MBP, but not GST. Anti-GST antibody recognised EG95–GST and GST, but only weakly recognised EG95–MBP.

### 3.3 Conformational and linear epitope recognition by Western blotting

**Fig. 5:**
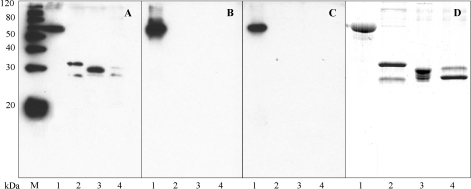
Western Blot. Recombinant proteins EG95–MBP (lane 1), EG95_4–74_ (lane 2), EG95_54–109_ (lane 3), and EG95_92-156_ (lane 4) probed with three antibody sources. Pooled sera from sheep vaccinated with EG95–GST (Panel A), polyclonal antibodies affinity purified to conformational epitopes of EG95 (anti-cEG95) (Panel B) and polyclonal antibodies affinity purified to peptide E100 (Panel C). A Coomasie stained SDS-PAGE displaying recombinant proteins.

EG95–MBP, EG95_4-74_, EG95_54-109_, and EG95_92-156_ were used to test recognition of conformational as well as linear epitopes of EG95. Pooled sera from sheep vaccinated with EG95–GST were shown by Western blot to recognise the entire molecule of EG95–MBP, as well as the truncated versions containing linear epitopes (Fig. 5). Anti-cEG95 antibodies reacted only with the entire EG95 molecule, but not with the truncated versions of EG95.

### 3.4 Selection of mimotopes for conformational epitopes of EG95 and GST

Twelve phage clones from the 20 mer library, and eight from the 12 mer library were selected from the final round of biopanning and shown by DNA sequencing to present different peptides. The only sequence homology to EG95 was found in peptide E14, which had five amino acid residues at its N terminal that aligned with residues at positions 115–122 of EG95. It is probable that this peptide mimics a linear epitope of EG95. The remainder of the peptide sequences bore no homology to the sequence of EG95 [39].

Eleven remaining selected phage clones were inhibited from binding to anti-cEG95 antibodies by the presence of EG95–MBP (data not shown). These 11 peptides were therefore considered mimics of conformational epitopes on EG95. One of these peptides, E100, was the mimotope chosen to be used in further affinity purification studies.

Three unique phage clones were selected from the biopanning of anti-GST antibodies. One peptide, G1, was the GST mimotope that was chosen to act as a control peptide in subsequent affinity purification studies.

### 3.5 Mimotope affinity purification

**Fig. 6:**
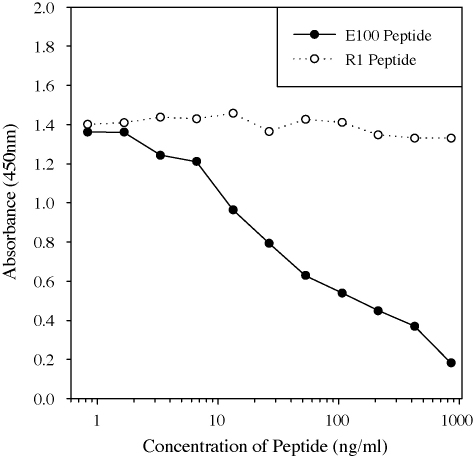
Inhibition of anti-E100 antibody binding to EG95 using E100 peptide. The concentration of anti-E100 antibody in each well was kept constant at 35 ng/ml and the concentration of free peptides E100 and R1 varied.

Antibodies were affinity purified from the sera of sheep vaccinated with EG95–GST using columns constructed using the EG95 mimotope (E100) as well as the GST mimotope (G1). A protein dot-blot assay was used to determine the specificity of the antibodies affinity purified from these columns. The results (Fig. 4) showed that antibodies affinity purified to E100 peptide were reactive to EG95–GST and EG95–MBP, but not to GST. By contrast antibodies affinity purified to G1 were reactive to EG95–GST and GST. Western blot showed that the anti-E100 antibodies did react with EG95–MBP, but did not react with the truncated versions of EG95 (Fig. 5). To determine whether or not the affinity purified anti-E100 antibodies represented a monospecific population the antibodies were subjected to an inhibition ELISA using peptide E100 and an irrelevant peptide R1. Anti-E100 antibodies were shown to be inhibited from binding to EG95–MBP by the addition of free peptide E100 in a concentration dependant manner (Fig. 6). The irrelevant peptide R1 was not able to inhibit the binding of E100 to EG95–MBP.

## 4 Discussion

In this study, we have reported the purification of polyclonal antibodies against the conformational determinants of the EG95 hydatid vaccine. Although there have been many reports of the purification of antibodies against a single epitope (monospecific purification), this appears to be the first report of purification of polyclonal antibodies specifically to conformational epitopes on a protein, at the exclusion of linear epitopes. These antibodies have then been used to successfully discover peptides that mimic these conformational epitopes.

Various methods have been advocated for the removal of antibodies with unwanted specificities in polyclonal samples that have been raised against recombinant antigens. One commonly used method is to add *E. coli* proteins to the serum solution [42] or to adsorb non-specific antibodies on nitrocellulose-bound antigens [43] to remove antibody specificities derived from exposure to bacterial proteins. Other negative or subtraction immunoaffinity methods have been described [44,45]. To date no method has been described to purify conformational epitopes from polyclonal serum at the exclusion of the linear specificities.

A two-stage affinity purification process was devised to collect polyclonal antibodies against conformational determinants on EG95. The first step involved the depletion of unwanted antibody specificities, and the second step was the affinity purification of antibodies against the protective epitopes.

This study used sepharose bound proteins for the depletion stage of the purification process. This involved the production of affinity purification column displaying GST and affinity purification columns displaying truncated versions of EG95. The GST affinity purification column was chosen because affinity depletion of antibodies specific to a fusion partner protein has been shown to be successful in eliminating antibodies with specificity to the fusion partner [46]. The columns that display the truncated versions of EG95 were used to affinity-deplete linear epitopes of EG95. These proteins do not contain the conformational epitopes of EG95 molecule [5]. Besides displaying linear epitopes of EG95, these proteins also contain the GST protein. When used as a fusion partner, GST has been assumed to display some epitopes that are not present when it is used without a fusion partner [47]. Thus the GST portion of these columns displaying truncated EG95 was expected to adsorb any antibodies with these specificities.

The affinity purified anti-cEG95 antibodies were found to be specific for EG95. In immuno-dot assays, the antibody reacted with EG95–MBP, EG95–GST, but not GST (Fig. 4). The anti-GST antibody specificities had been effectively removed from the sample. In addition the antibody solution did not react with the truncated proteins by Western blot (Fig. 5) indicating that the antibodies were specific for conformational epitopes. Additionally, the affinity purified anti-EG95 antibodies have been shown to bind to native oncosphere EG95 and to function in complement mediated oncosphere killing [39].

The production of polyclonal antibodies against conformational epitopes on particular antigens is especially applicable for use in affinity selection using RPPD libraries. Moreover, these antibodies may be useful agents in applications such as immunoblotting and immunocytochemistry. We have demonstrated that production of these antibodies is possible and shown that they are suitable for use with RPPD libraries. The purified antibodies are highly specific to the antigen of interest, with little cross reactivity to the fusion partner or linear epitopes on the antigen. The antibodies directed against linear determinants appear to have been reduced to such an extent that when used with RPPD libraries none of the selected peptides bore resemblance to the linear epitopes that dominated previous affinity selection experiments [11]. Further, we have shown that one of the selected peptide mimotopes is capable of affinity purifying monospecific antibodies that bind EG95 conformational epitopes. The technique of removing antibodies against linear epitopes described in this paper would be expected to be able to generate a target suitable for use with random peptide phage display libraries for any protein antigen where correct folding of the protein antigen is required for conferring immunity.
